# Leukemoid reaction and autocrine growth of bladder cancer induced by paraneoplastic production of granulocyte colony-stimulating factor – a potential neoplastic marker: a case report and review of the literature

**DOI:** 10.1186/1752-1947-8-147

**Published:** 2014-05-13

**Authors:** Anup Kasi Loknath Kumar, Megha Teeka Satyan, Jeffrey Holzbeierlein, Moben Mirza, Peter Van Veldhuizen

**Affiliations:** 1 Division of Hematology and Oncology, University of Kansas Medical Center, Kansas City, Kansas, USA; 2 Department of Urology, University of Kansas Medical Center, Kansas City, Kansas, USA

**Keywords:** Bladder cancer, Granulocyte colony-stimulating factor, Leukemoid reaction

## Abstract

**Introduction:**

Granulocyte colony-stimulating factor produced by nonhematopoietic malignant cells is able to induce a leukemoid reaction by excessive stimulation of leukocyte production. Expression of granulocyte colony-stimulating factor and its functional receptors have been confirmed in bladder cancer cells. *In vitro* studies have demonstrated that granulocyte colony-stimulating factor/receptor exhibits a high affinity binding and this biological axis increases proliferation of the carcinoma. Urothelial carcinoma of the bladder is rarely associated with a leukemoid reaction and autocrine growth induced by paraneoplastic production of granulocyte colony-stimulating factor. In the world literature, there have been less than 35 cases reported in the last 35 years. The clinicopathological aspects, biology, prognosis and management of granulocyte colony-stimulating factor-secreting bladder cancers are poorly understood.

**Case presentation:**

A 39-year-old Caucasian woman with an invasive high-grade urothelial carcinoma presented with hematuria and low-grade fevers. Laboratory tests revealed an elevated white blood cell count and absolute neutrophil count and an elevated 24-hour urine protein. Upon further evaluation she was found to have locally advanced high-grade urothelial carcinoma without nodal or distant metastasis. Her serum granulocyte colony-stimulating factor level was 10 times the normal limit. This led to the diagnosis of a paraneoplastic leukemoid reaction. Her white blood cell count immediately normalized after cystectomy but increased in concordance with recurrence of her disease. Unfortunately, she rapidly progressed and expired within 10 months from the time of first diagnosis.

**Conclusions:**

This is one of the few cases reported that illustrates the existence of a distinct and highly aggressive subtype of bladder cancer which secretes granulocyte colony-stimulating factor. Patients presenting with a leukemoid reaction should be tested for granulocyte colony-stimulating factor/receptor biological axis. Moreover, granulocyte colony-stimulating factor could be a potential neoplastic marker as it can follow the clinical course of the underlying tumor and thus be useful for monitoring its evolution. Neoadjuvant chemotherapy should be considered in these patients due to the aggressive nature of these tumors. With a better understanding of the biology, this autocrine growth signal could be a potential target for therapy in future.

## Introduction

Paraneoplastic leukemoid reaction is a type of paraneoplastic syndrome in which there is an elevation in the white blood cell count, predominantly neutrophils. Granulocyte colony-stimulating factor (G-CSF) secreted by nonhematopoietic malignant cells has been reported to be able to induce a leukemoid reaction through excessive stimulation of leukocyte production [1-3]. Urothelial carcinoma of the bladder is rarely associated with a leukemoid reaction, with less than 30 cases reported over the past 30 years, some of which tried to elucidate the role of G-CSF [1,3-7]. A previous study reported that 9.2% of bladder tumors are associated with high levels of G-CSF [8]. In addition, expression of G-CSF and functional G-CSF receptors (G-CSFR) has been demonstrated in bladder cancer cells [9]. *In vitro* studies have demonstrated that G-CSF/G-CSFR exhibit high affinity [1] binding and this biological axis increases proliferation in bladder cancer cells [1,9-11]. This autocrine mechanism of growth may be associated with aggressive tumor growth and adverse clinical outcomes [1,12]. Here, we present a rare case of a leukemoid reaction and autocrine growth of bladder cancer induced by paraneoplastic production of G-CSF. We review the literature on the main clinicopathological aspects of this important, but rare, condition and review the biology of G-CSF in bladder cancer and its implications for diagnosis, management, prognosis and future research.

## Case presentation

**Figure 1:**
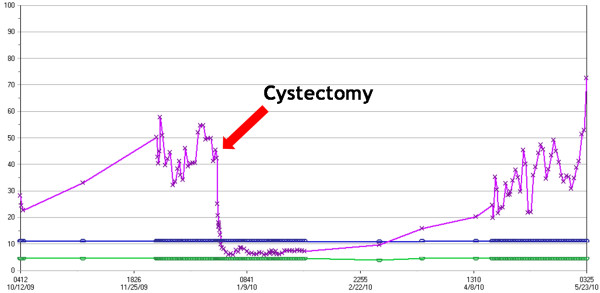
**Graphical representation of the trend of white blood cell counts before and after cystectomy (see arrow).** X axis represents time and Y axis represents white blood cell count in K/UL. Green and blue lines represent the normal range of white blood cell count. Pink line represents the trend in the patient’s white blood cell count.

**Figure 2:**
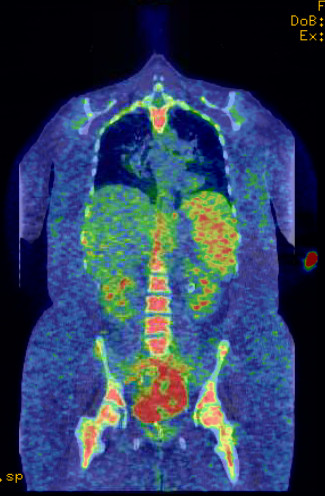
**Positron emission tomography scan showing metabolic activity only in the bladder, prior to cystectomy.** There is no evidence of regional or distant metastasis. There is slight increased activity in the skeletal system which is probably secondary to a marrow stenting agent.

**Figure 3:**
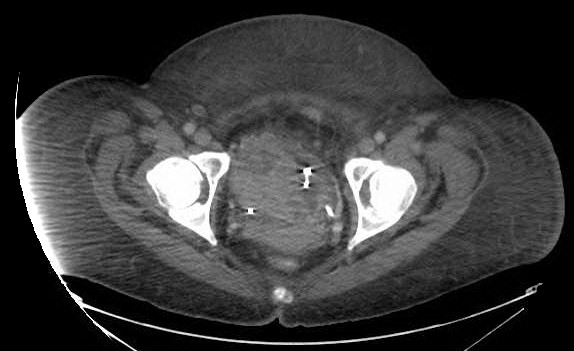
**Computed tomography scan of the pelvis showing large nodular bladder mass with heterogeneous enhancement, prior to cystectomy.** Exchange are most extensive along the right bladder wall though is also seen along the left bladder wall and fundus.

**Figure 4:**
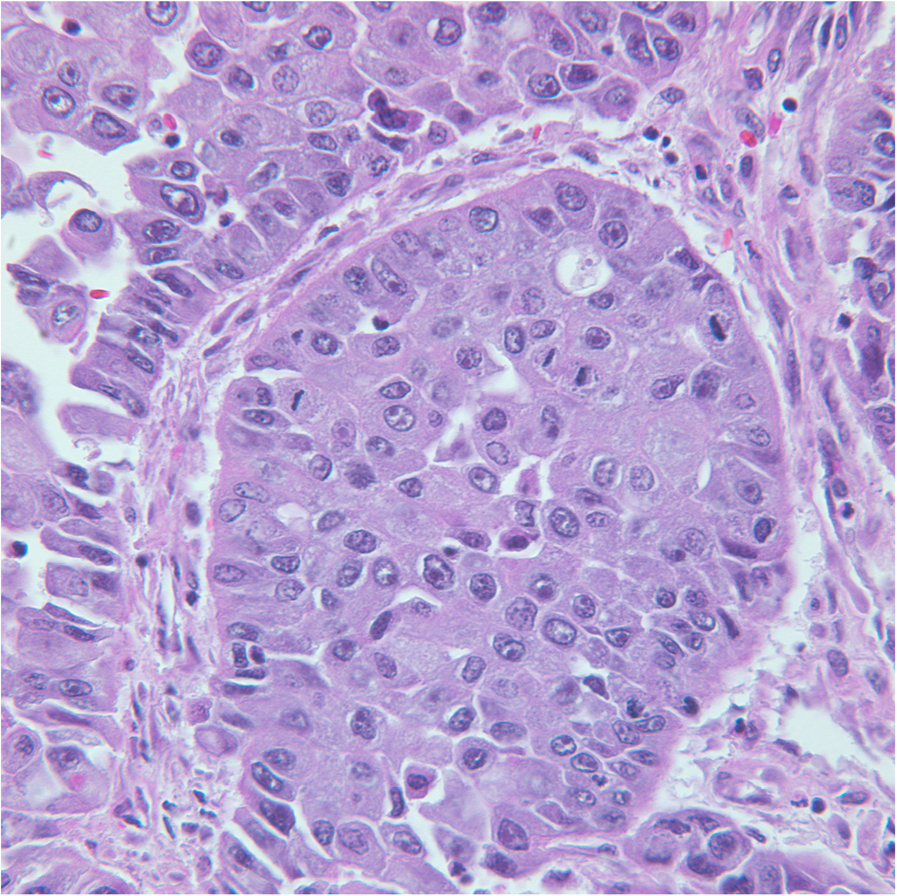
**Histological examination of the cystectomy specimen showing poorly differentiated high-grade transitional cell carcinoma.** (Hematoxylin and eosin section, original magnification×220).

A 39-year-old non-cigarette-smoking Caucasian woman with hypertension, type 2 diabetes, diabetic retinopathy and neuropathy was diagnosed with muscle invasive high-grade urothelial carcinoma with squamous and glandular differentiation and necrosis by transurethral resection of her bladder tumor 4 months prior to presentation to our hospital. Three weeks prior to presentation in our clinic she had noted gross hematuria, daily low-grade fevers, night sweats and weight gain of 32kg. A physical examination revealed that her temperature was 39°C (102.2°F), pulse 110 per minute, respiratory rate 20 per minute, and blood pressure 150/83mmHg. Her examination was only remarkable for progressive anasarca. Laboratory studies revealed elevated white blood cell (WBC) count and absolute neutrophil count (ANC; peak WBC 57.8K/UL, peak ANC 43.24K/UL), leukocyte alkaline phosphatase score 295, erythrocyte sedimentation rate (ESR) >140mm/hour, C-reactive protein (CRP) 29.5mg/dL, and antineutrophilic antibody (ANA) titer 320. She also had nephrotic range proteinuria with 24-hour urine protein of 14.65g and a serum creatinine of 3.27mg/dL (Table 1 and Figure 1). On admission, her urine grew greater than 100,000 *Aerococcus urinae* for which she was treated with a course of ceftriaxone. Repeat urine and blood cultures were negative but she had continued elevation of her WBC count. The results of anti-double stranded deoxyribonucleic acid (DNA) antibody, rheumatic factor, anti-SSA, anti-SSB, anti-glomerular basement membrane, myeloperoxidase, proteinase 3, cytoplasmic antineutrophil cytoplasmic antibody, perinuclear antineutrophil cytoplasmic antibody, hepatitis panel, and human immunodeficiency virus antibody tests were negative. Serum protein electrophoresis, creatine phosphokinase, quantitative immunoglobulin assay, complement levels and chest radiograph were all normal. Hence, symptoms, signs and laboratory studies were negative for an infectious etiology (including a tagged WBC scan) and autoimmune/rheumatological disease. A bone scan and positron emission tomography scan ruled out metastasis (Figure 2). A computed tomography (CT) scan showed a large nodular bladder mass (Figure 3). The fevers and night sweats were attributable to her malignancy and her weight gain/anasarca was related to her nephrotic syndrome. After her medical condition was optimized, she underwent a radical cystectomy, bilateral pelvic lymph node dissection, and ileal conduit urinary diversion along with total abdominal hysterectomy/bilateral salpingo-oophorectomy. Immediately after removal of her bladder tumor, her presenting symptoms and laboratory values improved remarkably. Fevers and night sweats completely resolved. Her WBC count was 6.8K/UL, ANC 5.05K/UL, ESR 45mm/hour, CRP 6.89mg/dL, ANA <80, 24-hours urine proteins of 5.25g, and serum creatinine 1.30mg/dL (Table 1). Histopathology revealed poorly differentiated high-grade transitional cell carcinoma invading the entire bladder wall and the myometrium of the lower uterine segment along with vascular invasion without metastasis. Her pathologic staging was pT4a,N0,M0 (Figure 4). In addition, to further evaluate her nephrotic syndrome, a renal ultrasound-guided biopsy was done. Her kidneys were of normal size. The biopsy showed diabetic nephropathy with extensive nodular glomeruli sclerosis, moderate interstitial fibrosis and mild focal chronic inflammation, mild tubular atrophy, and severe arteriolar hyalinosis. She responded to hemodialysis and her serum creatinine trended down to 1.30mg/dL with resolution of anasarca.

**Table 1:** Comparison of laboratory values before and after cystectomy

**Laboratory values**	**Before cystectomy**	**After cystectomy**
**Total white blood cell (K/UL)**	Peak 57.8	Peak 6.8
**Absolute neutrophil count (K/UL)**	Peak 43.24	Peak 5.05
**Leukocyte alkaline phosphatase score**	295	**–**
**Erythrocyte sedimentation rate (mm/hour)**	>140	45
**C**-**reactive protein (mg/dL)**	29.5	6.89
**Antineutrophilic antibody**	320	<80
**24-hour urine protein (g)**	14.65	5.25
**Serum creatinine (mg/dL)**	3.27	1.30

The patient refused adjuvant chemotherapy and returned 3 months following her cystectomy with an elevated WBC (peak WBC 45.3K/UL) and serum creatinine 1.90. A CT scan showed multiple pelvic masses with abdominal lymphadenopathy and a large right pelvic mass in close approximation to the area of the sigmoid colon causing obstruction. Her serum G-CSF level was 406.6pg/mL (normal <39.1pg/mL) confirming the diagnosis of paraneoplastic leukemoid reaction. She underwent a diverting colostomy to relieve symptoms of bowel obstruction. However due to poor performance status, renal failure and the development of a colovaginal fistula, she was placed on palliative care and expired soon thereafter.

## Discussion

This rare case of urothelial carcinoma of the bladder had several unusual features including young age at presentation, marked leukemoid reaction, elevated G-CSF levels (ten times the normal limit), nephrotic range proteinuria and very rapid progression of disease to death within 6 months of the onset of the leukemoid reaction. Urothelial carcinoma in this case showed squamous differentiation which has been reported to have elevated G-CSF levels [4-6]. A possible mechanism for development of leukemoid reaction and rapid metastatic spread of the tumor is that the high-grade urothelial carcinoma harbored both paraneoplastic production of G-CSF and expression of functional G-CSFR. This may have stimulated the bone marrow with increased myelopoiesis and leukemoid reaction. The stimulation of tumor growth may have been due to the expression of functional G-CSFR by the neoplastic cells, creating a positive feedback cycle. This mechanism has been demonstrated in *in vitro* studies [1,9,11]. Consistent with this mechanism, the above unusual features normalized after removal of the tumor (Table 1 and Figure 1). Similar post-surgical normalization of the leukemoid reaction and G-CSF has been previously observed in bladder cancer [5,13]. Three months later, with recurrence of tumor, there was elevated WBC and G-CSF. Hence, G-CSF could be used to follow the evolution of cancer and as a potential future prognostic marker. Of note, this case also exhibited nephrotic range proteinuria which improved after tumor removal, thereby clinically seemed to be paraneoplastic, but biopsy showed features of diabetic nephropathy. Minimal-change glomerulopathy associated with urothelial carcinoma has been rarely reported in the literature [14,15]. However, this may have been masked by her pre-existing diabetic nephropathy.

In the present case, as the tumor recurred and grew in her pelvic cavity, the concentration of serum G-CSF was elevated with an associated increase in her WBC count. These findings support the hypothesis that the bladder cancer cells producing G-CSF may grow more rapidly or that the G-CSF production exhibited by the tumor cells may promote the malignant progression. We thus undertook a comprehensive review of all studies and similar cases reported to date to explore the above hypothesis. It is also probable that the patient had micrometastatic disease, undetectable by currently existing diagnostic modalities, prior to surgery. Rapid development of metastases within 3 months after surgery may have been invigorated by G-CSF secretion and activation of the G-CSF/G-CSFR biological axis stimulating tumor growth.

A review by Turalic *et al.* showed that the average age of these patients was 70.1 years and the male to female ratio was 3:2. The WBC was as high as 181,800/mL (average 66,758/mL) and there was an average G-CSF level of 203pg/mL with a mean survival of 5.9 months following surgical therapy in these patients. However, those who received adjuvant chemotherapy reported survivals of >20 months [4]. An observational study in Japanese patients by Mizutani *et al.* in 1995 showed that 9.2% of bladder tumors had elevated G-CSF levels (mean G-CSF of 328), which positively correlated with an increase in grade and progression of stage of cancer, more so in patients with distant metastasis. Furthermore, patients with bladder cancer without elevated G-CSF had better disease-specific survival rates at 5 years follow-up when compared with those with an elevated level of serum G-CSF [8].

The mechanism responsible for overexpression of G-CSF in bladder cancer is yet to be clearly elucidated. Possibilities include rearrangement of the G-CSF gene occurring within one of the alleles [16] and intrinsic activation of nuclear factors that work on the promoter region of the G-CSF gene [17]. Treatment with phorbol ester, interleukin-1 beta, or interferon gamma increased the level of G-CSF, granulocyte-macrophage colony-stimulating factor, and macrophage colony-stimulating factor in an *in vitro* bladder cancer cell line study. This capability to respond to various stimuli implies that numerous regulatory pathways may be involved in the production of cytokines [18]. Although the mechanism of G-CSF gene overexpression requires further investigation, Chakraborty *et al.* conducted a series of *in vitro* studies which have shown that G-CSFR specifically signals for beta-1 integrin expression, adhesion and invasion of bladder cancer cells which could promote metastasis [19]. Furthermore, they demonstrated that the G-CSF/G-CSFR biological axis facilitated survival and growth of bladder cancer cells and stimulated STAT3-dependent survivin expression [11]. The histogenesis of transitional cell carcinoma of the bladder is uncertain, although different theories have been proposed. Some authors have suggested that a metaplastic phenomenon with various degrees of differentiation may be responsible for the malignant transitional cell G-CSF production [20]. In addition, this theory is supported further by the remarkable potential of the transitional epithelium and transitional cell carcinoma to differentiate into several lines [21]. The frequent finding of both squamous and glandular differentiation has long been observed in transitional cell carcinoma. Furthermore, the presence of neuroendocrine (small cell) differentiation has also been reported [22]. Hematopoietic differentiation of transitional cell carcinoma, resulting in the acquirement of G-CSF production and G-CSFR expression, is another possibility supported by our observations. Lastly, G-CSF may be released from the foci of tumor necrosis [7]. Hence, G-CSF could be a potential screening tool to predict aggressive cancer and hence pursue an early aggressive treatment modality.

G-CSF administration has been in vogue as an adjunct to the management of granulocytopenias, either with or without prior chemotherapy [23-26]. Subsequent studies have further confirmed the efficacy of G-CSF in conjunction with systemic chemotherapy for patients with advanced bladder cancer [27,28]. A few studies recommend G-CSF administration to enhance the tumor-specific cytotoxicity of chemotherapy [29,30], to allow dose intensification of chemotherapy [31-33] with low myelotoxicity and mucosal toxicity levels [31]. In contrast, Perez *et al.* reported a case of rapid clinical deterioration and leukemoid reaction after treatment of urothelial carcinoma with G-CSF along with chemotherapy [7]. Recombinant G-CSF may have both direct and indirect stimulatory effects on the growth of bladder cancer cells *in vitro*. It may stimulate the growth of the residual tumor cells after chemotherapy while it is being used clinically to combat myelosuppression induced by antitumor chemotherapy in bladder cancer [34]. Due to controversial reports, at this time, we can only advocate that all bladder cancers should be tested for the expression of G-CSFR before using G-CSF, because there is the possibility of growth stimulation by G-CSF if they have G-CSFR on their cell surfaces. With a better understanding of the biology, this autocrine growth signal could be a potential target for therapy in future.

## Conclusions

On the basis of this case experience and review of literature we conclude that there is a distinct and highly aggressive subtype of bladder cancer which secretes G-CSF and expresses G-CSFR inducing an autocrine growth cycle. Hence such patients presenting with leukemoid reaction should be tested for G-CSF/G-CSFR biological axis. Moreover, G-CSF could be a potential neoplastic marker as it can follow the clinical course of the underlying tumor and thus be useful for monitoring its evolution. Neoadjuvant chemotherapy should be considered as the aggressive nature of these tumors appears to overwhelm these patients before they receive adjuvant treatments. With a better understanding of the biology, this autocrine growth signal could be a potential target for therapy in future.

We are currently planning to conduct a preliminary study to identify patients with such bladder cancer, not only to estimate its prevalence but also to assess the variation in G-CSF levels in response to various therapies (surgery, chemotherapy, radiotherapy). In addition we will evaluate G-CSF as a prognostic marker and as a tool to predict a more aggressive biological phenotype of invasive bladder cancer. Future studies will be necessary to determine the regulatory effects of G-CSF on bladder cancer cells.

## Consent

Written informed consent was obtained from the patient’s next of kin for publication of this case report and any accompanying images. A copy of the written consent is available for review by the Editor-in-Chief of this journal.

## Abbreviations

ANA: Antineutrophilic antibody; ANC: Absolute neutrophil count; CRP: C-reactive protein; CT: Computed tomography; ESR: Erythrocyte sedimentation rate; G-CSF: Granulocyte colony-stimulating factor; G-CSFR: Granulocyte colony-stimulating factor receptor; WBC: white blood cell.

## Competing interests

The authors declare that we have no competing interests.

## Authors’ contributions

AKLK drafted the manuscript and prepared the figures. PVV, MM and JH reviewed and amended the manuscript. All authors read and approved the final manuscript.

## References

[B1] Tachibana M Miyakawa A Tazaki H Nakamura K Kubo A Hata J Nishi T Amano Y Autocrine growth of transitional cell carcinoma of the bladder induced by granulocyte-colony stimulating factor Cancer Res 1995 55 3438 3443 7542171

[B2] Demetri GD Griffin JD Granulocyte colony-stimulating factor and its receptor Blood 1991 78 2791 2808 1720034

[B3] Sato K Terada K Sugiyama T Masuda H Kakinuma H Kato T Granulocyte colony-stimulating factor produced by bladder carcinoma of a patient with leukemoid reaction did not affect proliferation of the tumor cells J Urol 1994 151 1687 1690 7514696 10.1016/s0022-5347(17)35345-4

[B4] Turalic H Deamant FD Reese JH Paraneoplastic production of granulocyte colony-stimulating factor in a bladder carcinoma Scand J Urol Nephrol 2006 40 429 432 10.1080/00365590600679350 17060092

[B5] Hirasawa K Kitamura T Oka T Matsushita H Bladder tumor producing granulocyte colony-stimulating factor and parathyroid hormone related protein J Urol 2002 167 2130 10.1016/S0022-5347(05)65104-X 11956459

[B6] McRae SN Gilbert R Deamant FD Reese JH Poorly differentiated carcinoma of bladder producing granulocyte colony-stimulating factor and parathyroid hormone related protein J Urol 2001 165 527 528 10.1097/00005392-200102000-00049 11176419

[B7] Perez FA Fligner CL Yu EY Rapid clinical deterioration and leukemoid reaction after treatment of urothelial carcinoma of the bladder: possible effect of granulocyte colony-stimulating factor J Clin Oncol 2009 27 e215 e217 10.1200/JCO.2009.22.4931 19786665

[B8] Mizutani Y Okada Y Terachi T Kakehi Y Yoshida O Serum granulocyte colony-stimulating factor levels in patients with urinary bladder tumour and various urological malignancies Br J Urol 1995 76 580 586 10.1111/j.1464-410X.1995.tb07782.x 8535676

[B9] Ohigashi T Tachibana M Tazaki H Nakamura K Bladder cancer cells express functional receptors for granulocyte-colony stimulating factor J Urol 1992 147 283 286 1370331 10.1016/s0022-5347(17)37214-2

[B10] Tachibana M Miyakawa A Uchida A Murai M Eguchi K Nakamura K Kubo A Hata JI Granulocyte colony-stimulating factor receptor expression on human transitional cell carcinoma of the bladder Br J Cancer 1997 75 1489 1496 10.1038/bjc.1997.254 9166942 PMC2223497

[B11] Chakraborty A Guha S Granulocyte colony-stimulating factor/granulocyte colony-stimulating factor receptor biological axis promotes survival and growth of bladder cancer cells Urology 2007 69 1210 1215 10.1016/j.urology.2007.02.035 17572226

[B12] Sires C Neely S Skinner D Leukemoid reaction in a patient with bladder and prostatic cancer J Urol 1986 135 366 367 3944874 10.1016/s0022-5347(17)45641-2

[B13] Stav K Leibovici D Siegel YI Lindner A Leukemoid reaction associated with transitional cell carcinoma Isr Med Assoc J 2002 4 223 224 11908271

[B14] Martinez-Vea A Panisello JM Garcia C Cases A Torras A Mayayo E Carrera M Richart C Oliver JA Minimal-change glomerulopathy and carcinoma. Report of two cases and review of the literature Am J Nephrol 1993 13 69 72 10.1159/000168592 8322844

[B15] Cases A Torras A Lens XM Revert L Minimal-change nephrotic syndrome associated with an undifferentiated urothelial carcinoma Med Clin (Barc) 1986 86 436 3713303

[B16] George DD Griffin JD Granulocyte colony stimulating factor and its receptor. *Blood* 1991, 78:2791–2795 (cited by Mizutani Y, Okada Y, Terachi T, Kakehi Y, Yoshida O: Serum granulocyte colony-stimulating factor levels in patients with urinary bladder tumour and various urological malignancies Br J Urol 1995 76 580 586 10.1111/j.1464-410X.1995.tb07782.x 8535676

[B17] Nishizawa M Tsuchiya M Watanabe-Fukunaga R Nagata S Multiple elements in the promoter of granulocyte colony-stimulating factor gene regulate its constitutive expression in human carcinoma cells J Biol Chem 1990 265 5897 5902 1690717

[B18] Steube KG Meyer C Tachibana M Murai M Drexler HG Bladder carcinoma cell line KU-19-19-derived cytokines support proliferation of growth factor-dependent hematopoietic cell lines: modulation by phorbol ester, interferon-gamma and interleukin-1 beta Biochem Biophys Res Commun 1998 242 497 501 10.1006/bbrc.1997.8002 9464244

[B19] Chakraborty A White SM Guha S Granulocyte colony-stimulating receptor promotes beta1-integrin-mediated adhesion and invasion of bladder cancer cells Urology 2006 68 208 213 16844458 10.1016/j.urology.2006.01.046

[B20] Iles RK Chard T Human chorionic gonadotropin expression by bladder cancers: biology and clinical potential J Urol 1991 145 453 458 1705292 10.1016/s0022-5347(17)38367-2

[B21] Grammatico D Grignon DJ Eberwein P Shepherd RR Hearn SA Walton JC Transitional cell carcinoma of the renal pelvis with choriocarcinomatous differentiation. Immunohistochemical and immunoelectron microscopic assessment of human chorionic gonadotropin production by transitional cell carcinoma of the urinary bladder Cancer 1993 71 1835 1841 10.1002/1097-0142(19930301)71:5<1835::AID-CNCR2820710519>3.0.CO;2-5 8448745

[B22] Grignon DJ Ro JY Ayala AG Shum DT Ordonez NG Logothetis CJ Johnson DE Mackay B Small cell carcinoma of the urinary bladder. A clinicopathologic analysis of 22 cases Cancer 1992 69 527 536 10.1002/1097-0142(19920115)69:2<527::AID-CNCR2820690241>3.0.CO;2-7 1309435

[B23] Gabrilove JL Jakubowski A Scher H Sternberg C Wong G Grous J Yagoda A Fain K Moore MA Clarkson B Oettgen HF Alton K Welte K Souza L Effect of granulocyte colony-stimulating factor on neutropenia and associated morbidity due to chemotherapy for transitional-cell carcinoma of the urothelium N Engl J Med 1988 318 1414 1422 10.1056/NEJM198806023182202 2452983

[B24] Crawford J Ozer H Stoller R Johnson D Lyman G Tabbara I Kris M Grous J Picozzi V Rausch G Smith R Gradishar W Yahanda A Vincent M Stewart M Glaspy J Reduction by granulocyte colony-stimulating factor of fever and neutropenia induced by chemotherapy in patients with small-cell lung cancer N Engl J Med 1991 325 164 170 10.1056/NEJM199107183250305 1711156

[B25] Morstyn G Campbell L Souza LM Alton NK Keech J Green M Sheridan W Metcalf D Fox R Effect of granulocyte colony stimulating factor on neutropenia induced by cytotoxic chemotherapy Lancet 1988 1 667 672 2895212 10.1016/s0140-6736(88)91475-4

[B26] Ohno R Tomonaga M Kobayashi T Kanamaru A Shirakawa S Masaoka T Omine M Oh H Nomura T Sakai Y Hirano M Yokomaku S Nakayama S Yoshida Y Miura AB Morishima Y Dohy H Niho Y Hamajima N Takaku F Effect of granulocyte colony-stimulating factor after intensive induction therapy in relapsed or refractory acute leukemia N Engl J Med 1990 323 871 877 10.1056/NEJM199009273231304 1697646

[B27] Aso Y Akaza H Effect of recombinant human granulocyte colony-stimulating factor in patients receiving chemotherapy for urogenital cancer. Urological rhG-CSF Study Group J Urol 1992 147 1060 1064 1372661 10.1016/s0022-5347(17)37468-2

[B28] Miyanaga N Akaza H Shimazui T Ohtani M Koiso K The effect of dose intensity on M-VAC therapy for advanced urothelial cancer Cancer Chemother Pharmacol 1994 35 Suppl S5 S8 7527735 10.1007/BF00686910

[B29] Ohigashi T Granulocyte-colony stimulating factor enhances the cytotoxic effects of methotrexate to bladder cancer cells in vitro Keio J Med 1990 39 254 260 10.2302/kjm.39.254 1704940

[B30] Akaza H Fukushima H Koiso K Aso Y Enhancement of chemotherapeutic effects by recombinant human granulocyte colony-stimulating factor on implanted mouse bladder cancer cells (MBT-2) Cancer 1992 69 997 1002 10.1002/1097-0142(19920215)69:4<997::AID-CNCR2820690428>3.0.CO;2-U 1370920

[B31] Viens P Gravis G Bladou F Lechevallier E Baume D Camerlo J Cowen D Coulange C Serment G Resbeut M Maraninchi D Impact of recombinant human granulocyte colony stimulating factor on dose intensity and toxicity of three cycles of methotrexate, vinblastine, doxorubicin and cisplatin in patients with previously untreated urothelial bladder carcinoma Eur Cytokine Netw 1996 7 395 399 8954183

[B32] Pronzato P Bertelli G Bruna F Tani F Vaira F Vanoli M Vigani A Intensified M-VEC chemotherapy with G-CSF support as outpatient treatment for advanced bladder cancer Anticancer Res 1997 17 2325 2327 9216710

[B33] de Wit R Overview of bladder cancer trials in the European Organization for Research and Treatment Cancer 2003 97 2120 2126 10.1002/cncr.11288 12673705

[B34] Shameem IA Kurisu H Matsuyama H Shimabukuro T Naito K Direct and indirect effects of recombinant human granulocyte-colony stimulating factor on *in vitro* colony formation of human bladder cancer cells Cancer Immunol Immunother 1994 38 353 357 10.1007/BF01517203 7515769 PMC11038229

